# Intentional Fallers with Complex Pelvic and Acetabular Fractures Do Not Have worse Radiological and Functional Outcomes than Accidental Fallers

**DOI:** 10.1155/2022/8292345

**Published:** 2022-01-10

**Authors:** Yi-Hsun Yu, Ying-Chao Chou, Bo-Yan Yeh, Yung-Heng Hsu, I-Jung Chen, Lien-Chung Wei

**Affiliations:** ^1^Department of Orthopedic Surgery, Musculoskeletal Research Center, Chang Gung Memorial Hospital and Chang Gung University 33302, Tao-Yuan, Taiwan; ^2^Department of Chinese Acupuncture and Traumatology, Center of Traditional Chinese Medicine, Chang Gung Memorial Hospital, Linkou, Taiwan; ^3^Department of Addiction Psychiatry, Tao-Yyuan Mental Hospital, Ministry of Health and Welfare, Taiwan

## Abstract

Individuals who fall from heights of ≥6 m can suffer from complex pelvic and acetabular fractures. The extent to which an intentional fall correlates with prognosis and outcome after osteosynthesis is unclear. Therefore, we aimed to investigate the clinical outcomes of fallers with pelvic and acetabular fractures after osteosynthesis and compare the radiological and functional outcomes between intentional and accidental fallers. We retrospectively reviewed 49 fallers who fell from heights of ≥6 m, developed pelvic and acetabular fractures, survived after resuscitation, and completed surgical treatment between 2014 and 2017. Fallers were divided into intentional and accidental fallers. Sixteen patients were intentional fallers, whereas the rest of the patients were accidental fallers. Psychiatric counseling was provided to each of the intentional fallers during follow-up. All intentional fallers had preexisting mental disorders, and the most common diagnosis was adjustment disorder. The group of intentional fallers predominantly comprised females that had a higher injury and new injury severity scores and longer hospital stays. However, early loss of fixation (<3 months) and functional outcomes (Merle d'Aubigné and Majeed hip scores at 6- and 12-month follow-ups) did not significantly differ between intentional and accidental fallers. We found that intentional fallers with pelvic and acetabular fractures may have more severe combined injuries compared to accidental fallers. However, the radiological and functional outcomes of the intentional fallers after osteosynthesis were not inferior to those of the accidental fallers with the implementation of well-designed surgical protocols and individualized physical and mental rehabilitation programs.

## 1. Introduction

The optimal treatment of pelvic and acetabular fractures remains challenging for orthopedic surgeons, especially for multiplanar unstable pelvic and complex acetabular fractures. Unstable pelvic and acetabular fractures are usually caused by high-energy traumas such as high-speed motor vehicular accidents, falls from heights, and crush injuries [1]. Among the different mechanisms of unstable pelvic and acetabular fractures, the mechanism of falling from great heights always causes great instability of the pelvic ring or multiple levels of injury of the acetabulum, resulting in treatment difficulties and unpredictable prognoses [2–4].

There is a high incidence of unstable pelvic and acetabular fractures in patients who fall from great heights [2, 3]. Most patients fall accidentally; however, some fall from jumping intentionally. According to the literature, intentional fallers tend to have preexisting mental disorders and often fail to comply with medical orders due to mood changes, psychological abnormalities, and a history of drug abuse [5–8], which contributes to an unpredictable prognosis after undergoing orthopedic procedures.

Previous studies mostly focused on epidemiology, resuscitation outcomes, and quality of life assessment of patients with mental disorders who experienced pelvic fractures after attempting to commit suicide by jumping [6–8]. However, it is not known whether their functional performance after injury was only related to the injury or to other factors related to their mental disorders. Therefore, the primary outcome evaluation of the current study is aimed at investigating the functional and radiological outcomes of patients with unstable pelvic and acetabular fractures caused by an intentional fall and comparing their clinical outcomes to those of accidental fallers who had similar injuries.

## 2. Materials and Methods

### 2.1. Study Design, Participants, and Data Collection

The current study retrospectively reviewed medical and imaging records of patients with unstable pelvic and acetabular fractures who were admitted between 2014 and 2017. The inclusion criteria were patients with unstable pelvic and acetabular fractures due to a fall from heights of ≥6 m who underwent osteosynthesis during the study period. The exclusion criteria were (1) age below 18 years, (2) the height of fall < 6 m, (3) conservative treatment for the pelvic ring injury and acetabular fracture, and (4) incomplete radiological and functional follow-ups. Furthermore, enrolled patients were divided into the following two groups according to the etiology of all their clinical parameters and outcome comparisons: intentional fallers (I group) and accidental fallers (A group). The review process was approved by the Institutional Review Board of Chang Gung Medical Foundation (IRB no. 201900247B0). All methods in this study were carried out in accordance with relevant guidelines and regulations. Additionally, informed consent was obtained from all subjects.

### 2.2. Patient Resuscitation Protocol

Patients were transferred to the emergency department of a tertiary referral trauma center either from the trauma scene or local hospitals. The resuscitation protocol was standardized and individualized, following the Acute Trauma Life Support guidelines and the hospital's resuscitative algorithm for pelvic fracture. Injured patients were admitted to regular wards or intensive care units after they were hemodynamically stabilized during the resuscitation stage. Osteosynthesis was performed once the patient's medical condition was stabilized, and the patient was suitable for undergoing anesthesia and surgery.

### 2.3. Fracture Classification and Operative Protocol

The current study adapted the AO/OTA classification for pelvic fractures, Judet-Letournel classification for acetabular fractures, and the Denis and Roy-Camille classification for sacral fractures. There were different strategies used in the treatment of pelvic ring injury and acetabular fracture. For pelvic ring injury, the posterior pelvic ring was always reduced and fixed at first. For the fractures with vertical instability with a displacement of affected hemipelvis > 10 mm, osteosynthesis would be performed by open reduction and internal fixation (ORIF) such as spinopelvic osteosynthesis or *trans*-iliac plating. Closed reduction and internal fixation (CRIF) with percutaneous fixation by iliosacral screw or *trans*-iliac-*trans*-sacral screw would be performed for those with less instability or those who could not tolerate osteosynthesis in the prone position. If a disrupted anterior pelvic ring was indicated for surgery, a sequential osteosynthesis (ORIF or CRIF) would be performed during the same anesthesia or a few days later, depending on the patient's condition.

However, for acetabular fracture, anterior approaches were first considered. Anterior acetabular approaches, such as the ilioinguinal approach, anterior intrapelvic approach, and pararectus, would be chosen depending on the patients' concomitant injuries. Commonly, an acetabular fracture involving posterior columns, such as transverse type, T type, anterior column plus hemitransverse, and associated columns, could be well reduced and fixed through anterior approaches. For those fractures of the posterior column with residual instability or gap or posterior wall, osteosynthesis would be performed subsequently through the posterior approach (Kocher-Lagenbeck approach).

### 2.4. Management and Rehabilitation Protocols after Osteosynthesis

Postoperative rehabilitation protocols were individualized according to the patient's medical condition or concomitant injuries. In general, toe-touch weight-bearing could be allowed 6 to 8 weeks after the index surgery, and crutch- or walker-free ambulation could be started 6 weeks after toe-touch ambulation. The definition of loss of fixation include the following: (1) implant breakage, (2) mobilization of fractured fragments > 5 mm in any direction compared to previous X-rays, and (3) radiolucent line around the implanted instrumentation before the fracture was united.

Mechanical prophylaxis for venothromboembolism was routinely applied using compression socks for 12 weeks. No routine prophylactic practice for heterotrophic ossification following surgery was provided. For intentional fallers, routine psychiatric consultations were performed to confirm their diagnosis, stabilize their mental condition, and prevent further suicide attempts.

Detailed records of outpatient clinic follow-up were documented during the 12 months follow-up. Functional outcome evaluations were conducted by using questionnaires for modified Merle d'Albinague score and Majeed hip score at 6 and 12 months. Radiological evaluations were evaluated at 2-, 4-, 12-, and 24-week interval postoperatively.

### 2.5. Statistical Analysis

Statistical analysis was conducted using SPSS 18.0 (SPSS Inc., Chicago, IL, USA). Continuous variables were presented as the mean and standard deviation. The normal distribution of the cohort was tested using the Shapiro-Wilk test. We compared the preoperative and perioperative variables between accidental and intentional fallers using the nonparametric Mann–Whitney *U*-test. Categorical variables were reported as frequencies, and we compared the data of the two groups by using Fisher's exact test. A two-tailed *P* value of 0.05 was considered significant. In addition, a post hoc power analysis was performed using G^∗^Power software (version 3.1.9.4; Franz Paul, University Kiel, Germany) to determine the power of primary outcomes of the difference in trauma scores, early loss of follow-up, early loss of fixation, and functional score evaluations between the two groups.

## 3. Results

### 3.1. Study Population and Characteristics

During the study period, a total of 848 patients with fractures involving the pelvis and acetabulum were admitted to the emergency department of our hospital. The incidence of index fracture resulting from fall injury (>6 m) was 21.9% (186/848). Forty-nine patients (26.3%, 49/186) were treated surgically. The complete demographic data for the 49 patients are presented in Table 1.

Among the enrolled patients, 16 were injured from intentional falls, including seven men and nine women, with a median age of 36.9 years (range: 18–92 years). All 16 patients had documented preexisting mental disorders. The majority of the patients had adjustment disorders (*n* = 9, 56.2%), whereas the others were diagnosed with schizophrenia, substance abuse disorder, and mixed type mental disorders. Ten patients (81.2%) were diagnosed with pelvic ring and sacral injuries, whereas the remaining patients had complex fractures involving the pelvic ring, sacrum, and acetabulum. The types of osteosynthesis are shown in Table 2. ORIF was the most common osteosynthesis for both anterior and posterior pelvic ring injuries, consisting of 19 and 9, respectively. There were various surgical approaches for the reduction and fixation of anterior acetabular fractures, whereas Kocher-Lagenbeck approach was the only approach for posterior acetabular fractures. Four patients (25%) never returned to the outpatient clinic department for follow-up evaluation after they received complete treatment and were discharged from the hospital. For the remaining 12 patients who had regular follow-ups, three patients (25%) showed early loss of reduction or broken implants within 3 months after osteosynthesis. However, no patients underwent revision surgery even when it was highly recommended.

### 3.2. Comparison between Intentional and Accidental Fallers

A comparison of the radiological and functional outcomes between patients whose injuries were caused by intentional falls (I group) and those whose injuries were caused by accidental falls (A group) is presented in Table 3. The I group was significantly more likely to be female (post hoc power, 80.3%), with a higher frequency of mental disorders (post hoc power, 100%), higher injury severity scores (ISS) (post hoc, 72.8%), higher new injury severity score (NISS) (post hoc power, 64.7%), higher abbreviated injury scale (AIS)-chest scores (post hoc power, 62.6%), higher AIS-abdomen scores (post hoc power, 41.9%), and longer hospital stays (post hoc power, 54.2%).

Results of outcome evaluations and comparisons are shown in Table 4. Radiological outcome evaluations were performed by X-ray examination during each clinical follow-up. Two patients in the I group who completed at least 3 months of follow-up showed a dislodged or broken implant (3/12, 25%). In the A group, three out of the 31 patients showed broken implants using the same assessment parameters (9.7%), but no significant difference (*P* = 0.325, Fisher's exact test) was found between the two groups.

Functional outcome evaluations were performed at 6 and 12 months after the index surgery. Overall, 30 patients (11 and 19 patients in I and A groups, respectively) completed functional outcome evaluations. Regardless of the time point of functional evaluation, there was no significant difference between the two groups, although there were more favorable functional outcomes for patients in the A group.

## 4. Discussion

The present study investigated the radiological and functional outcomes for intentional fallers after osteosynthesis of pelvic and acetabular fractures and compared their clinical outcomes with those of accidental fallers. The results revealed that all intentional fallers had preexisting mental disorders. Additionally, intentional fallers were predominantly female and had higher ISS, NISS, AIS-chest, and AIS-abdomen scores and longer hospital stays. However, early loss of fixation and functional outcomes at the 6- and 12-month follow-ups did not significantly differ between the intentional and accidental fallers.

Falls, regardless of etiology, can impact socioeconomic development. Accidental fallers are usually of working age; hence, they make financial contributions to their families and society [2]. Functional disability following a fall injury may impact their work productivity. However, intentional fallers, also called suicide jumpers, are a common public health concern affecting all levels of society [8–16]. In Taiwan, suicide is the second leading cause of death among young people aged 15–24 years, the third cause among those aged 25–44 years, and the 12th cause in the general population [17]. Regardless of the reason for the fall, all survivors in our case study fell from a height of >6 m, hitting architectural ledges or falling onto hard-impact sites. Thus, they had higher ISS and NISS scores compared to patients who were the subject of previous reports [2, 8].

According to the literature, suicide risk is 10 times higher in patients with a psychiatric disorder than those without such disorders; at least one psychiatric disorder was reported in 60% to 98% of individuals who committed or attempted suicide [18–20]. The most common mental disorder in this group is depressive disorder (35–80%), followed by schizophrenia (10%) and dementia or delirium (5%), which is consistent with the results of our study [21]. Prevention of suicide attempts is also a key concern in reducing the morbidity and mortality from suicide. In our study, all intentional fallers were reported to have preexisting mental disorders. A routine psychiatric consultation was performed for each intentional faller with a preexisting mental disorder, and treatment with medication and mental health consultation was conducted to prevent a recurrence. Fortunately, more than half of the intentional fallers had high compliance with medical orders, and there was no recidivism during the follow-up period. Routine psychiatric counseling may have contributed to the similar functional outcomes observed for patients in the two groups.

Postosteosynthesis patients should strictly follow medical advice and rehabilitation protocols in the early postoperative stage to prevent fixation failure, especially those with unstable pelvic and acetabular fractures. Patients with mental disorders are thought to have poorer compliance with medical orders [2, 22, 23]. Thus, we expected that intentional fallers with pelvic and acetabular fractures would have a higher incidence of fixation failure than accidental fallers. Although 25% of the intentional fallers experienced fixation failure at an early stage, no significant difference was found between the I and A groups (*P* = 0.345). This finding might indicate that a secure fracture fixation was made for all patients, allowing them to start with gait training as soon as possible. However, we noticed a 9.7% loss of reduction and fixation in accidental fallers during our follow-up. This finding could be explained by their eagerness to return to the activities of daily living as well as a desire to return to work in order to earn and also to retain their jobs.

Several factors were associated with functional outcomes following orthopedic surgeries, including characteristics of fractures, quality of reduction and fixation, complications, rehabilitation protocol, and patient compliance [23–28]. During the study period, the principles, implants, surgical techniques, and physical therapies used in treating pelvic and acetabular fractures were similar for all patients. Our data revealed that the severity of injury and length of hospital stay were statistically higher and longer in the I group than in the A group. However, at the 6- and 12-month evaluations, there was no difference in functional outcomes between the two groups.

Although we made efforts to avoid bias, there were still some limitations in the current study. First, a relatively small population was enrolled in this study, and only 73.5% of the patients completed radiological and functional outcomes. Nevertheless, two important findings of our study are that the functional outcomes of the intentional fallers were not inferior to those of the accidental fallers if the fractures were managed appropriately and that there was no recidivism during the follow-up period. Second, we classified the patients by the etiology of their injuries according to their self-reported history; the true number of intentional fallers with mental disorders may be underreported, thereby affecting the results of the analysis. Lastly, the concept of functional evaluation for pelvic and acetabular fractures may be different. We found that 22.4% (11/49) of the cohort were diagnosed with combined pelvic and acetabular fractures. Therefore, we used two evaluation scales (Merle d'Aubigné hip score and Majeed hip score) to evaluate their functional performance during activities of daily living. In the future, a larger sample size should be enrolled, so that different fractures (i.e., pelvis or acetabulum) can be analyzed separately. In addition, different evaluation tools, such as the Short Form-36 and other hip functional scores, should be applied to comprehensively evaluate patients.

## 5. Conclusions

In conclusion, intentional fallers with pelvic and acetabular fractures may display more severe combined injuries compared to accidental fallers. With the implementation of well-designed surgical protocols and individualized physical and mental rehabilitation programs, the radiological and functional outcomes for intentional fallers could be comparable to those of accidental fallers.

## Figures and Tables

**Table 1:** Demographic distribution of 49 patients with pelvic and acetabular fractures who underwent osteosynthesis.

All patients	49
Sex	
Male	35
Female	14
Age	43.5 ± 15.3
Injury mechanism	
Intentional	16
Accidental	33
Injury severity score	16.6 ± 10.2
New injury severity score	19.8 ± 9.9
Fracture site and its classification	
Pelvis	31
61-B	16
61-C	15
Acetabulum	23
Anterior column	2
Transverse	1
Transverse+posterior wall	1
T type	2
Anterior column plus posterior hemitransverse	6
Associated both columns	10
Sacrum	17
Dennis zone I	8
Dennis zone II	9
Roy-Camille type 1	2
Roy-Camille type 2	7
Roy-Camille type 3	1
Intensive care unit stay (days)	3.6 ± 5.4
Hospital stay (days)	21.2 ± 12.2
Follow-up (months)	15.3 ± 12.5

**Table 2:** Types of osteosynthesis in treating pelvic ring injuries and acetabular fractures.

	Pelvis				Acetabulum	
	Anterior ring		Posterior ring			
ORIF	Pfannestiel	10	Spinopelvic osteosynthesis	7	Ilioinguinal	9
	Ilioinguinal	4	*Trans*-iliac plate	2	AIP+lateral window	7
	Lateral window	4			Pararectus approach	1
	Pararectus	1			Extended ilioinguinal	1
					Kocher-Lagenbeck	8
CRIF	Anterior column screw	2	Iliosacral screw	7		
			*Trans*-iliac*-trans*-sacral screw	7		

ORIF: open reduction and internal fixation; CRIF: closed reduction and internal fixation; AIP: anterior intrapelvic approach.

**Table 3:** Clinical comparison between intentional and accidental fallers.

	Intentional	Accidental	*P* value
Sex			0.006
Male	7	28	
Female	9	5	
Age	36.9 ± 21.1	46.6 ± 10.6	0.099
ISS	21.9 ± 10.3	14.1 ± 9.2	0.01
NISS	24.3 ± 9.0	17.5 ± 9.7	0.023
AIS-head	0.6 ± 1.3	0.5 ± 1.0	0.357
AIS-face	0.3 ± 0.8	0.2 ± 0.5	0.915
AIS-chest	2.4 ± 1.7	1.2 ± 1.6	0.020
AIS-abdomen	1.7 ± 1.3	1.0 ± 1.2	0.049
AIS-extremity	2.6 ± 0.7	2.5 ± 0.6	0.942
Mental disorder	16	0	<0.001
Intensive care unit stay	4.6 ± 4.6	3.1 ± 5.7	0.053
Hospital stay	26.5 ± 12.6	18.6 ± 10.8	0.018
Time to surgery	6.94 ± 3.6	8.13 ± 4.3	0.329
Follow-up duration	19.9 ± 15.0	13.7 ± 11.4	0.154
Follow-up of <3 months	25% (4/16)	27.3 (9/33)	1.000
Unstable pelvic fracture	81.3% (13/16)	54.5% (18/33)	0.165
Unstable acetabular fracture	45.4 (5/11)	77.4 (24/31)	0.0006

**Table 4:** Primary outcome comparison between the two groups.

	Intentional	Accidental	*P* value
Loss of fixation (<3 months)	3/12 = 25.0%	3/31 = 9.7%	0.325
Functional follow-up			
Merle d'Aubigné hip score			
6 months	9.0 ± 4.5 (*n* = 11)	10.0 ± 3.7 (*n* = 19)	0.084
12 months	11.9 ± 4.5 (*n* = 11)	13.4 ± 3.6 (*n* = 19)	0.475
Majeed hip score			
6 months	51.0 ± 17.8 (*n* = 11)	55.3 ± 17.2 (*n* = 19)	0.090
12 months	68.3 ± 17.4 (*n* = 11)	72.6 ± 17.8 (*n* = 19)	0.094

## Data Availability

All data generated or analyzed during this study are included in this published article.
